# Feasibility of home administration of nebulised interferon ß-1a (SNG001) for COVID-19: a remote study

**DOI:** 10.3399/BJGPO.2023.0089

**Published:** 2023-09-20

**Authors:** Nick A Francis, Phillip D Monk, Jacqueline Nuttall, Thomas Oliver, Catherine Simpson, Jody L Brookes, Victoria J Tear, Angela G Thompson, Toby N Batten, Marcin Mankowski, Thomas MA Wilkinson

**Affiliations:** 1 Primary Care Research Centre, Faculty of Medicine, University of Southampton, Aldermoor Health Centre, Southampton, UK; 2 Synairgen Research Ltd, Southampton, UK; 3 Southampton Clinical Trials Unit, University of Southampton and University Hospital Southampton NHS Foundation Trust, Southampton, UK; 4 Quinn Clinical Limited, Merseyside, UK; 5 Veramed, Twickenham, London, UK; 6 tranScrip Ltd, Wokingham, UK; 7 NIHR Southampton Biomedical Research Centre, Clinical and Experimental Sciences, University of Southampton, Southampton, UK

**Keywords:** SARS-CoV-2, COVID-19, home monitoring, feasibility studies

## Abstract

**Background:**

Effective therapeutics given early to high-risk ambulatory patients with coronavirus disease 2019 (COVID-19) could improve outcomes and reduce overall healthcare burden. However, conducting site visits in non-hospitalised patients, who should remain isolated, is problematic.

**Aim:**

To evaluate the feasibility of a purely remote (virtual) study in non-hospitalised patients with COVID-19; and the efficacy and safety of nebulised recombinant interferon-β1a (SNG001) in this setting.

**Design & setting:**

Randomised, double-blind, parallel-group study, which was conducted remotely.

**Method:**

Eligible patients aged ≥65 years (or ≥50 years with risk factors) with COVID-19 and not requiring hospital admission were recruited remotely. They were randomised to SNG001 or placebo once-daily via nebuliser for 14 days. The main outcomes were assessments of feasibility and safety, which were all conducted remotely.

**Results:**

Of 114 patients treated, 111 (97.4%) completed 28 days of follow-up. Overall compliance to study medication was high, with ≥13 doses taken by 89.7% and 92.9% of treated patients in the placebo and SNG001 groups, respectively. Over the course of the study, only two patients were hospitalised, both in the placebo group; otherwise there were no notable differences between treatments for the efficacy parameters. No patients withdrew owing to an adverse event, and a similar proportion of patients experienced on-treatment adverse events in the two treatment groups (64.3% and 67.2% with SNG001 and placebo, respectively); most were mild or moderate and not treatment-related.

**Conclusion:**

This study demonstrated that it is feasible to conduct a purely virtual study in community-based patients with COVID-19, when the study included detailed daily assessments and with medication administered via nebuliser.

## How this fits in

Recruiting patients with COVID-19 in the community into studies is made challenging by the need for isolation, and most studies have been conducted in the hospital setting. However, only a small proportion of patients with COVID-19 require hospitalisation. The current study demonstrated that it is feasible to conduct a purely virtual study in non-hospitalised patients with COVID-19, with the study including detailed daily assessments and with study medication administered via nebuliser. Future, larger, purely virtual studies are planned.

## Introduction

One challenge with conducting clinical studies, especially during the severe acute respiratory syndrome coronavirus 2 (SARS-CoV-2) pandemic, is investigative site attendance, in particular when recruiting non-hospitalised patients with COVID-19. Many COVID-19 studies have therefore been conducted in hospitals. However, only a small proportion of patients with COVID-19 require hospitalisation. Early treatment of ambulatory patients could potentially improve outcomes, with an earlier return to productivity and reduced healthcare resource utilisation. Novel studies conducted in the community setting are therefore needed.

Interferon-β is a naturally occurring protein produced as an immediate local response to viral infection, resulting in antiviral protein production, limiting viral replication.^1–3^ SARS-CoV-2 suppresses interferon-β release,^4,5^ facilitating viral spread throughout the respiratory tract; the host innate immune response can potentially be enhanced by inhaling interferon-β.^3^ SNG001 is a unique formulation of recombinant interferon-β1a that contains few excipients and has near-neutral pH, making it suitable for inhaled administration via nebuliser.

Two studies have evaluated the effect of SNG001 in hospitalised patients with COVID-19. The first was a phase II study conducted early in the pandemic, in which patients receiving SNG001 were more likely to improve and recovered more rapidly than those receiving placebo.^6^ The second, a phase III study, suggested that SNG001 may prevent progression to severe disease, although the primary endpoint was not met.^7^

The phase II study included a second cohort who did not require hospitalisation at recruitment, and who are the subject of this article. This cohort followed an innovative home-based study design, with all interactions conducted remotely. The overall aims were to evaluate feasibility of such a study in patients with COVID-19 who were inhaling nebulised medication, and to evaluate the efficacy and safety of SNG001 in this setting.

## Method

**Figure 1.**
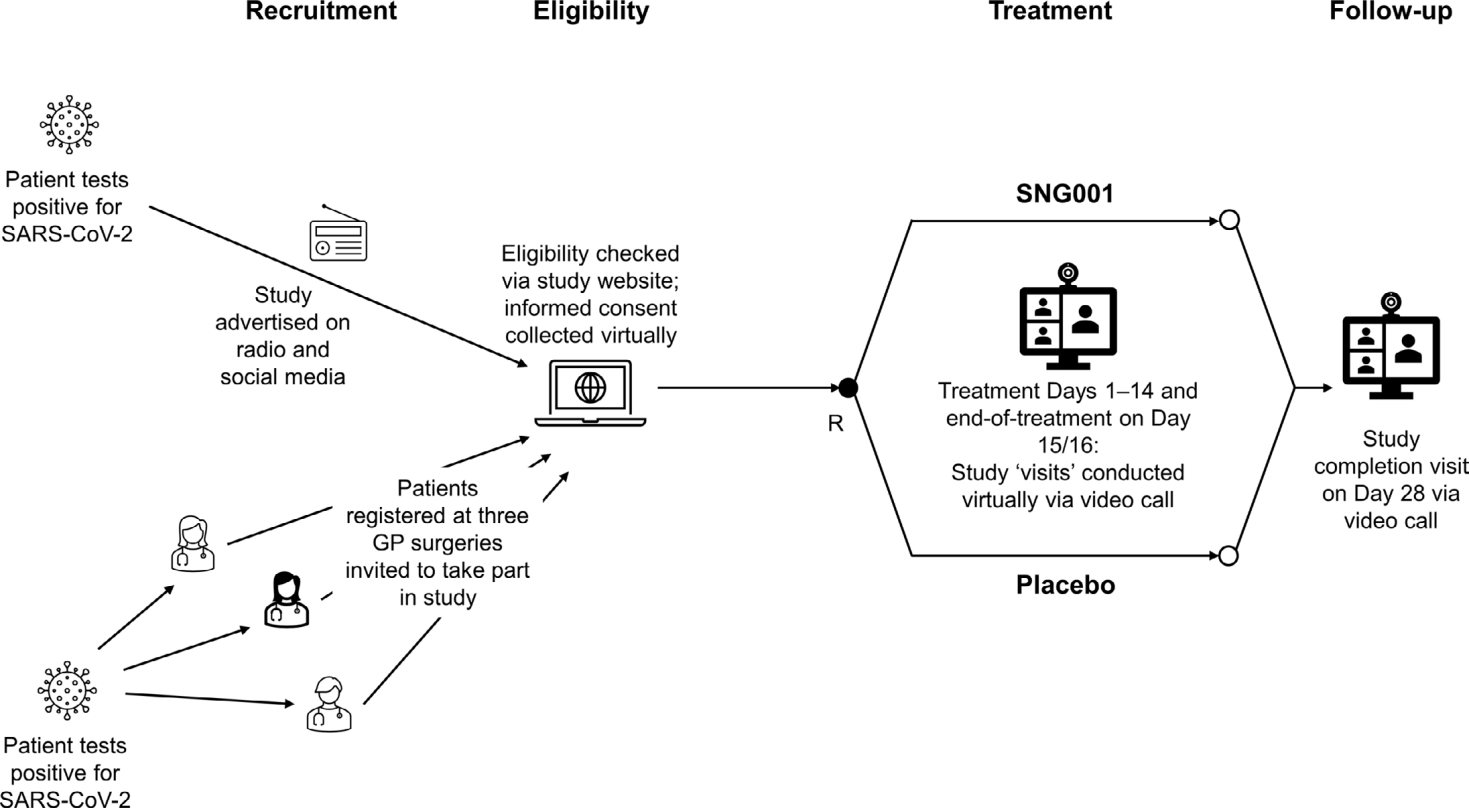
Study schematic. SARS-CoV-2 = severe acute respiratory syndrome coronavirus 2. R = randomisation.

This randomised, double-blind, placebo-controlled study was conducted in England, Scotland, and Wales. Patients were recruited remotely using a study website (accompanied by radio advertising and social media), and three general practices (Figure 1). The website questionnaire determined if they had COVID-19 symptoms (high temperature, new continuous cough, loss of or change to sense of smell or taste) starting in the previous 7 days. Eligible patients were ≥65 years of age (or ≥50 with a risk factor; see Supplementary file, *Methods*) who did not require hospitalisation, and tested positive for SARS-CoV-2 in the prior 7 days. Full inclusion and exclusion criteria are in the Supplementary file (*Methods)*. All study ‘visits’ were conducted virtually by nurses or doctors using video calls, with informed consent collected via the study website before any study-related procedure.

Patients were randomised to inhale SNG001 or placebo once-daily via nebuliser for 14 days. They were sent study medication, nebuliser, pulse oximeter, thermometer, and other consumables, and were trained in the use of the nebuliser via video calls. Once assessed as being competent in nebuliser use, they were able to take trial medication before or after the video calls.

Prior to the first dose, daily for the 14-day treatment period, and on days 15 or 16 (that is, end of treatment) and 28, the following were assessed via video call, with questionnaires read out by a study nurse: World Health Organization Ordinal Scale of Clinical Improvement ([WHO OSCI]; rated 0–8; see Supplementary file, *Methods*);^8^ Breathlessness, Cough and Sputum Scale ([BCSS]; see Supplementary file, *Methods*);^9^ COVID-19 symptoms (see Supplementary file, *Methods*); self-reported overall wellness (1–10); any health services contact (post-baseline only); self-reported recovery (yes or no); and vital signs. EuroQol 5-dimension 5-level (EQ-5D-5L) was assessed at baseline and on days 7, 15-or-16, and 28.

The only protocol amendments that impacted patients were extensions to the SARS-CoV-2 molecular assay timelines for practical reasons (initially increased from 24 to 96 hours before entry, and then to 7 days), and the addition to the list of COVID-19 symptoms of loss of or change to sense of smell or taste.

### Interventions

Patients were centrally randomised to treatment according to a prespecified randomisation schedule generated by an independent statistician, with investigators, patients, and sponsor blinded to treatment using matched placebo, with study medication presented in pre-filled syringes (Supplementary file, *Methods*). Study drug was administered via the I-neb or Solo/Ultra nebuliser, with randomisation schedule stratified by device.

### Outcomes

The main outcomes were assessments of feasibility and safety, and to inform future study design. The primary endpoint was change in WHO OSCI during the dosing period, in terms of hospitalisation, time-to-first recovery (WHO OSCI score ≤1 with no rebound >1 at any subsequent visit up to day 28, inclusive), recovery at each post-baseline assessment, improvement at each post-baseline visit, time-to-first improvement by ≥1 (with no return to baseline up to day 28, inclusive), odds for better outcome at each visit, worsening by ≥1 (analysed after day 3), worsening to ≥2, and the number of days with score ≥2 (Supplementary file, *Methods*). Secondary endpoints included the following: time-to-clinical improvement (temperature ≤37.8°C and absent or mild COVID-19 symptoms); proportion of patients with a recovery to no limitation of activities (WHO OSCI <2) on day 7 and at treatment end; time to self-reported recovery; self-reported daily overall wellness rating; quality-of-life (EQ-5D-5L); daily BCSS score (including disaggregated scores); health services contact; and consumption of antibiotics.

### Statistical methods

The study was not formally powered. It was anticipated that 120 patients (60 per treatment arm) would be sufficient to evaluate feasibility.

As no previous clinical WHO OSCI data had been collected in a relevant population when the study was designed, the most appropriate way of analysing WHO OSCI score was unknown. Consequently, multiple analyses of the primary endpoint were conducted to support future study design. There was no hierarchy across analyses, and none of the endpoints were adjusted for multiplicity. Informal hypothesis testing was at the 5% α-level. All analyses were adjusted for device and baseline WHO OSCI score (categorised as ≤1 or ≥2), and were done over the treatment period (defined as 16 days: the 14-day dosing period and the end-of-treatment visit on day 15 or 16) or at each individual visit. WHO OSCI improvement was analysed with an ordered logistic regression model assuming proportional odds. Times to WHO OSCI recovery and improvement were analysed using Cox proportional hazard models. Time to WHO OSCI recovery only included patients with a baseline WHO OSCI score ≥2, and the WHO OSCI baseline covariate was excluded. Sustained WHO OSCI recovery, hospitalisation, WHO OSCI worsening, and worsening to WHO OSCI ≥2 were also analysed with logistic regression models. Worsening to WHO OSCI ≥2 only included patients with a baseline score <2 and the baseline covariate was excluded. The number of days at WHO OSCI ≥2, excluding the covariate for WHO OSCI at baseline, was analysed using an analysis of covariance. A last-observation-carried-forward approach was used to impute missing WHO OSCI data for all non-time-to-event analyses. All analyses were done with SAS (version 9.4), with data from the two nebulisers pooled for each treatment group. The nebuliser was not expected to influence treatment efficacy; however, device was included in relevant statistical analyses as a covariate. Statistical methods for the secondary endpoints are in the Supplementary file, *Methods*.

The intention-to-treat (ITT) population, used for the efficacy analyses, was all randomised patients who received at least one dose of study medication. The safety population, used for the safety analyses, was the same as the ITT population.

## Results

**Figure 2.**
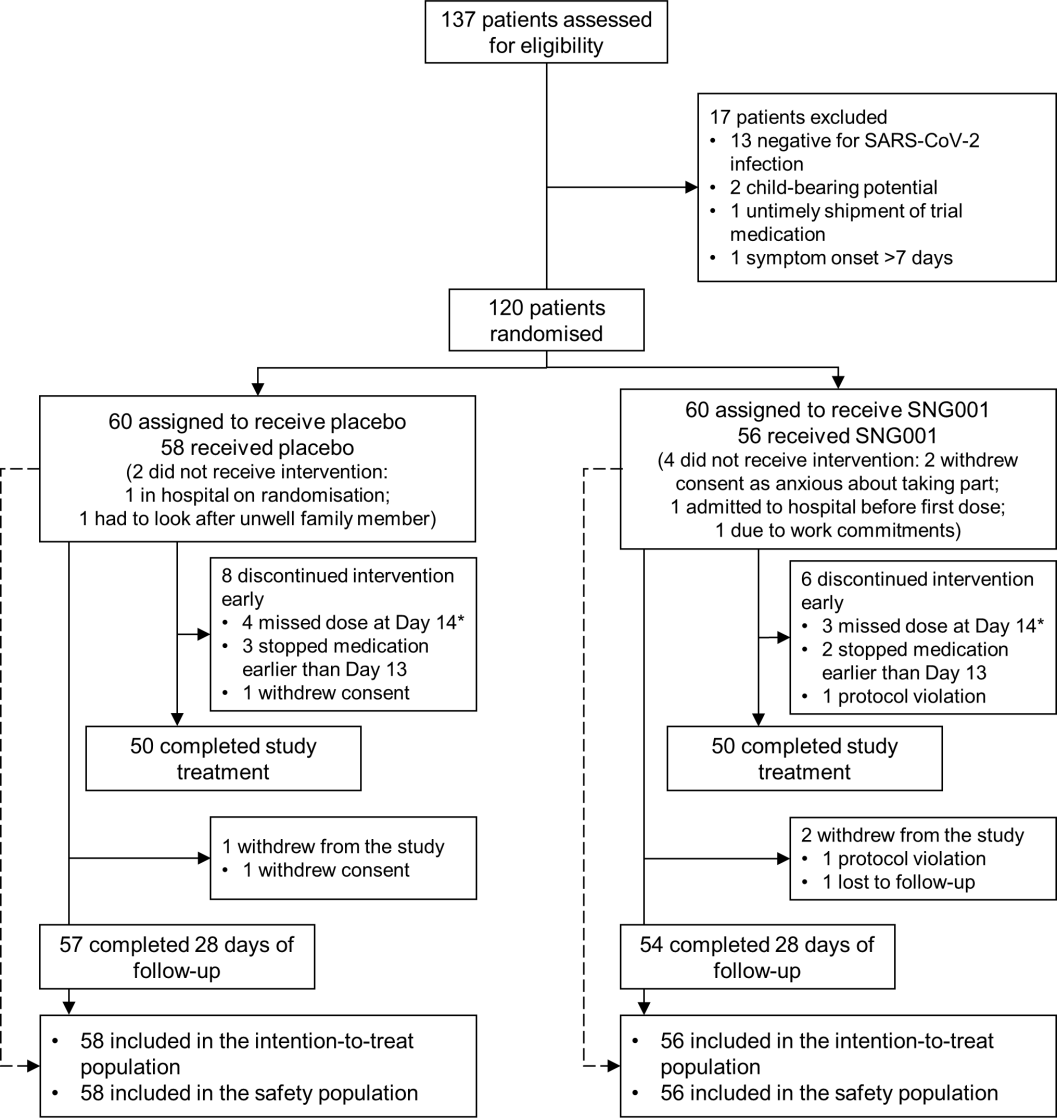
Patient disposition. *All seven patients who missed the day 14 dose had a technical difficulty with one of the earlier doses (for example, spillage), which resulted in the patient using two doses on one day. This then meant that the patient did not have the day-14 dose available, and so received 13 doses.

The study was conducted between 30 May 2020 and 23 April 2021. Of 137 patients screened, 120 were randomised, with 58 and 56 receiving at least one dose of placebo and SNG001, respectively, 57 (98.3%) and 54 (96.4%) of whom completed 28 days of follow-up (Figure 2). As prespecified, the two patients randomised to placebo and four randomised to SNG001 who did not receive study medication were excluded from all analyses. Demographics and disease characteristics were broadly similar in the two groups, although there was a higher proportion of male patients in the SNG001 group (Table 1). Overall compliance to study medication was high, with ≥13 doses taken by 89.7% and 92.9% of patients in the placebo and SNG001 groups, respectively.

**Table 1.** Demographic and baseline characteristics

Characteristic, *n* (%) unless stated	Placebo (*n* = 58)	SNG001 (*n* = 56)
Mean age, years (SD); range	61.5 (7.27); 50–79	60.1 (8.07); 50–84
Sex, male	23 (39.7%)	33 (58.9%)
Race, White	55 (94.8%)	53 (94.6%)
Mean end-of-treatment BMI, kg/m^2^ (SD)	29.4 (5.36)	29.6 (6.44)
Any comorbidity	48 (82.8%)	47 (83.9%)
Severity of disease at baseline		
WHO OSCI 1: No limitation of activities	20 (34.5%)	24 (42.9%)
WHO OSCI 2: Limitation of activities (ambulatory)	38 (65.5%)	32 (57.1%)
Mean (SD) BCSS total score	3.5 (1.98)	3.7 (2.21)
Breathlessness	1.1 (1.13)	1.1 (1.15)
Cough	1.9 (0.93)	2.0 (1.01)
Sputum	0.5 (0.78)	0.6 (0.84)

BMI = body mass index. BCSS = Breathlessness, Cough and Sputum Scale. WHO OSCI = World Health Organization Ordinal Scale of Clinical Improvement.

### Efficacy

At baseline, all patients had WHO OSCI scores of 1 or 2, with mean BCSS total scores of 3.5 and 3.7 in the placebo and SNG001 groups, respectively (Table 1), indicating limited day-to-day impact of COVID-19. During the treatment period, only two patients were hospitalised according to WHO OSCI, both receiving placebo; this endpoint was therefore not analysed. Of the 38 patients receiving SNG001 with WHO OSCI >1 at baseline, 20 (52.6%) met the recovery definition, compared with *n* = 15/32 (46.9%) receiving placebo, with no difference in time-to-first recovery (SNG001 versus placebo hazard ratio [HR] 0.84 [95% confidence interval {CI} = 0.43 to 1.65]; *P* = 0.62), or the proportion recovering at any timepoint (Supplementary Figure S1).

There were also no significant differences between groups in the proportion with WHO OSCI improvement ≥1 (*n* = 17/56 [30.4%] with SNG001 versus *n* = 24/58 [41.4%] with placebo), or time-to-first improvement by ≥1 (HR 0.75 [95% CI = 0.40 to 1.40]; *P* = 0.36), and the only difference in the proportional odds analysis for better outcome was on day 15-or-16 (SNG001 versus placebo odds ratio [OR] 0.46 [95% CI = 0.22 to 0.97]; *P* = 0.040). The proportions of patients with WHO OSCI worsening ≥1 (after day 3) were similar in both groups (19.6% versus 15.5%; OR 0.89 [95% CI = 0.27 to 2.95]; *P* = 0.85), as was the proportion with worsening from <2 at baseline to ≥2 at any visit (21.4% versus 17.2%; OR 0.96 [95% CI = 0.28 to 3.24]; *P* = 0.94). The LS mean number of days with WHO OSCI ≥2 was 5.70 with SNG001 versus 5.38 with placebo (*P* = 0.71).

When analysed in patients with a fever or symptoms at baseline, *n* = 23/54 patients (42.6%) receiving SNG001 improved versus *n* = 24/51 patients (47.1%) receiving placebo, with similar times-to-clinical improvement in the two groups (HR 0.71 [95% CI = 0.39 to 1.28]; *P* = 0.25). The proportions of patients recovering to no limitation of activities (WHO OSCI <2) on days 7 and 15-or-16 were similar in the two groups (day 7: 51.8% versus 48.3%, *P* = 0.852; day 15-or-16: 66.1% versus 70.7%, *P* = 0.69).

Sustained recovery was self-reported by 32.1% versus 43.1% of patients in the SNG001 versus placebo groups, with similar times to recovery (HR 0.62 [95% CI = 0.34 to 1.14]; *P* = 0.12), and no differences between groups in wellness (Supplementary Figure S2) or EQ-5D-5L (Supplementary Figure S3). BCSS breathlessness and cough scores improved from baseline over the treatment period in both groups (Supplementary Figures S4 and S5); changes from baseline in sputum scores were small (Supplementary Figure S6). BCSS total score also improved from baseline in both groups, with a significantly greater improvement with placebo on days 13 and 14 (*P* < 0.05; Supplementary Figure S7). There were no differences between groups in health services contact (Supplementary Figure S8) or antibiotic consumption (25.0% with SNG001 versus 20.7% with placebo).

### Safety

None of the patients who started study medication withdrew owing to an adverse event. A similar proportion of patients experienced on-treatment adverse events in the two groups (64.3% and 67.2% with SNG001 and placebo, respectively), with most events mild or moderate in severity and not considered related to study treatment (Table 2 and Supplementary Table S1). The only serious adverse event in more than one patient in either group was COVID-19 pneumonia, in two patients in the SNG001 group, both assessed as unrelated to treatment. There were no clinically meaningful findings in vital signs or other safety assessments.

**Table 2.** Adverse events (safety population)

Adverse event type	Placebo, *n* (%) (*n* = 58)	SNG001, *n* (%)(*n* = 56)
Any adverse event	46 (79.3%)	38 (67.9%)
Any adverse event related to study treatment	15 (25.9%)	15 (26.8%)
Any severe adverse event	6 (10.3%)	3 (5.4%)
Any serious adverse event	2 (3.4%)	4 (7.1%)
Any fatal adverse event	0	0
Any adverse event leading to withdrawal from the study	0	0

## Discussion

### Summary

Overall, this study confirmed the feasibility of conducting a purely virtual study in patients with non-hospitalised COVID-19, in which recruitment, screening, randomisation, and regular visits were all conducted remotely (with many recruited during lockdown). This meant that there was no need for patients to leave home, or for the study team to visit participants. Perhaps as a consequence, a high proportion of patients completed 28 days of follow-up (97.4%), with only one patient lost to follow-up. Furthermore, nearly all completed the assessments each day of the treatment period. Although effective in generating efficacy and safety data from these virtual visits, the study was not powered for these outcomes and no consistent or marked SNG001–placebo differences were apparent. Finally, the current study, in patients with milder disease who did not require hospitalisation, confirmed the good overall safety and tolerability profile of SNG001 observed in previous studies in patients hospitalised with COVID-19,^6,7^ with no patients who started study medication withdrawing owing to an adverse event, and most events mild or moderate in severity and not considered related to study treatment. The established safety and tolerability profile of SNG001 was one reason that the current study was possible. An intervention with potential safety concerns or less characterised safety would have been less qualified for use in a purely remote study.

### Strengths and limitations

The main strength of the study is the high level of patient participation, both in terms of the proportion completing follow-up, and the number completing the assessments. The inclusion criteria (≥65 years of age, or ≥50 years of age and with a known risk factor) were designed to recruit a population at risk of severe disease. In the event, only two patients (both with placebo) were hospitalised according to WHO OSCI during the treatment period, with BCSS scores at baseline indicating a limited COVID-19 impact. Recruitment of patients with milder COVID-19 than expected is the main limitation of the study, impacting the efficacy evaluations given there was not a significant clinical problem to solve. In addition, patients were required to be knowledgeable in the use of technology, including having email accounts and being able to use video calling. This may have excluded some patients with lower technological literacy or with digital accessibility limitations, and who may have been willing to participate in face-to-face research. Finally, six recruited patients (four with SNG001, two with placebo) did not receive any study medication, and were therefore excluded from the analyses. Only two of these (both in the SNG001 group) withdrew consent, and as this was not only before administration of the study medication, but before any regular study evaluations, the authors do not believe that this reflects actual feasibility or acceptability of either the intervention or the trial.

### Comparison with existing literature

In a number of previous studies in non-hospitalised patients with COVID-19, the incidence of subsequent hospitalisation was substantially higher than the 3.4% receiving placebo (and none receiving SNG001) in the current study. For example, in a study that was part of the ACTIV-2 platform trial, one patient (0.9%) receiving SNG001 was hospitalised, compared with 6.4% receiving placebo.^10^ Similarly, in the remdesivir PINETREE study, 5.3% of patients in the placebo group were hospitalised by day 14;^11^ and in a study evaluating the efficacy of oral nirmatrelvir, 6.2% of patients receiving placebo were hospitalised;^12^ whereas in a study evaluating the efficacy of oral molnupiravir, 14.1% of patients receiving placebo were hospitalised or died.^13^

### Implications for research and practice

The current study demonstrated that it is feasible to conduct a purely virtual study in community-based patients with COVID-19, when the study included detailed daily assessments and with study medication administered via nebuliser. Future larger, purely virtual studies are planned. The study also demonstrated that patients can be trained in the use of nebulised medication remotely, making use of video technology. This is consistent with the day-to-day work of UK-based GPs, with some face-to-face consultations replaced with video or telephone-based interactions, especially in patients who are unable to leave home (either for medical reasons or owing to COVID-induced lockdown).

## Trial registration number

NCT04385095

## Data

The data analysed and presented in this study are available from the corresponding author on reasonable request, providing the request meets local ethical and research governance criteria after publication. Patient-level data will be anonymised and study documents will be redacted to protect the privacy of trial participants.
